# Diagnostic Value of Subjective Memory Complaints Assessed with a Single Item in Dominantly Inherited Alzheimer's Disease: Results of the DIAN Study

**DOI:** 10.1155/2015/828120

**Published:** 2015-04-02

**Authors:** Christoph Laske, Hamid R. Sohrabi, Mateusz S. Jasielec, Stephan Müller, Niklas K. Koehler, Susanne Gräber, Stefan Förster, Alexander Drzezga, Felix Mueller-Sarnowski, Adrian Danek, Mathias Jucker, Randall J. Bateman, Virginia Buckles, Andrew J. Saykin, Ralph N. Martins, John C. Morris, Dominantly Inherited Alzheimer Network (DIAN)

**Affiliations:** ^1^German Center for Neurodegenerative Diseases (DZNE), 72076 Tübingen, Germany; ^2^Section for Dementia Research, Department of Cellular Neurology, Hertie Institute for Clinical Brain Research and Department of Psychiatry and Psychotherapy, University of Tübingen, 72076 Tübingen, Germany; ^3^Department of Psychiatry and Psychotherapy, University of Tübingen, 72076 Tübingen, Germany; ^4^Centre of Excellence for Alzheimer's Disease Research and Care, School of Medical Sciences, Edith Cowan University, Perth, WA 6027, Australia; ^5^School of Psychiatry and Clinical Neurosciences, University of Western Australia, Nedlands, WA 6009, Australia; ^6^Division of Biostatistics, Washington University School of Medicine, St. Louis, MO 63108, USA; ^7^Department of Neurodegeneration, Hertie Institute for Clinical Brain Research, University of Tübingen, 72076 Tübingen, Germany; ^8^Department of Nuclear Medicine & TUM Neuroimaging Center (TUM-NIC), Klinikum Rechts der Isar, Technical University Munich, 80333 Munich, Germany; ^9^Department of Nuclear Medicine, University of Cologne, 50937 Cologne, Germany; ^10^Deutsches Zentrum für Neurodegenerative Erkrankungen, 81377 München, Germany; ^11^Neurologische Klinik und Poliklinik, Ludwig-Maximilians-Universität, 81377 München, Germany; ^12^Department of Cellular Neurology, Hertie Institute for Clinical Brain Research, University of Tübingen, 72076 Tübingen, Germany; ^13^Department of Neurology, Knight Alzheimer's Disease Research Center, Washington University School of Medicine, St. Louis, MO 63108, USA; ^14^Indiana Alzheimer Disease Center, Indiana University School of Medicine, Indianapolis, IN 46202, USA; ^15^Department of Neurology, Washington University in St. Louis, MO 63108, USA

## Abstract

*Objective*. We examined the diagnostic value of subjective memory complaints (SMCs) assessed with a single item in a large cross-sectional cohort consisting of families with autosomal dominant Alzheimer's disease (ADAD) participating in the Dominantly Inherited Alzheimer Network (DIAN). *Methods*. The baseline sample of 183 mutation carriers (MCs) and 117 noncarriers (NCs) was divided according to Clinical Dementia Rating (CDR) scale into preclinical (CDR 0; MCs: *n* = 107; NCs: *n* = 109), early symptomatic (CDR 0.5; MCs: *n* = 48; NCs: *n* = 8), and dementia stage (CDR ≥ 1; MCs: *n* = 28; NCs: *n* = 0). These groups were subdivided by the presence or absence of SMCs. *Results*. At CDR 0, SMCs were present in 12.1% of MCs and 9.2% of NCs (*P* = 0.6). At CDR 0.5, SMCs were present in 66.7% of MCs and 62.5% of NCs (*P* = 1.0). At CDR ≥ 1, SMCs were present in 96.4% of MCs. SMCs in MCs were significantly associated with CDR, logical memory scores, Geriatric Depression Scale, education, and estimated years to onset. *Conclusions*. The present study shows that SMCs assessed by a single-item scale have no diagnostic value to identify preclinical ADAD in asymptomatic individuals. These results demonstrate the need of further improvement of SMC measures that should be examined in large clinical trials.

## 1. Introduction

Alzheimer's disease (AD) is currently conceptualized as progressing in three stages including (1) preclinical AD, (2) early symptomatic AD that has been characterized as mild cognitive impairment (MCI) due to AD, and (3) dementia due to AD [1]. After publication of these research criteria in 2011, the research focus has moved to the preclinical stage, as it is considered a highly promising target for future early intervention [2]. Thus, identification of early symptoms characterizing the preclinical stage of AD is highly needed.

Although interest is increasing in subjective memory complaints (SMCs), it remains uncertain due to controversial findings whether they are meaningful or not with respect to the diagnosis of preclinical sporadic AD. Some studies have found that SMCs are associated with an increased risk of developing AD [3, 4], while other studies have found that they are not predictive of cognitive impairment or future cognitive decline [5–7] and may correlate better with depressive symptoms than with cognitive symptoms [8–11]. The terms “subjective memory/cognitive complaints/decline” have been used in different ways throughout the last 40 years. In earlier years, “subjective” meant “reporting memory concerns by the subject in any stage of cognitive impairment” [12–16]. In most current literature, the term “subjective cognitive decline” is used for defining a state in the preclinical stage of sporadic AD and “subjective” means “in absence of objective impairment” [17]. In the present study, we use the term SMC because individuals are asked concerning their memory decline and not concerning other cognitive functions. In principle, the term SMC can be used at different stages of cognitive impairment, as done in the present study, as far as in which stage it is examined is defined (e.g., “SMC in preclinical AD,” “SMC in MCI,” or “SMC in dementia”).

Autosomal dominant AD (ADAD) is a rare form of AD, caused by mutations in genes encoding presenilin-1 (PSEN1), presenilin-2 (PSEN2), or amyloid precursor protein (APP) and leading to young onset dementia. The opportunity to observe trajectories of changes in biological and psychological parameters of dementia-related processes is explored by the Dominantly Inherited Alzheimer Network (DIAN), studying ADAD in a large sample of pedigrees with early onset forms of dementia. Baseline characteristics and biomarker findings in this cohort are on record [18, 19]. A previous study with carriers of the PSEN1 E280A mutation identified a pre-MCI stage with already existing memory complaints [20]. However, this study focused on mutation carriers (MCs) and did not describe the frequency of SMCs in noncarriers (NCs) and the diagnostic value of SMCs in the different stages of ADAD.

At present the diagnostic value of SMCs in the different stages of ADAD is unclear. The aim of the current study was to address this open question by examining the frequency and diagnostic accuracy of SMCs in MCs and NCs of ADAD participating in the DIAN study in different clinical stages.

## 2. Materials and Methods

All aspects of the study were approved by the institutional review boards for each of the participating sites in DIAN. All participants provided written informed consent.

### 2.1. Participants

The baseline sample of 183 MCs and 117 NCs participants (first-degree relatives of MCs) of the DIAN study underwent clinical assessment of cognitive status by means of the Clinical Dementia Rating (CDR) scale [21, 22] and was consecutively divided into preclinical (asymptomatic; CDR 0), early symptomatic (CDR 0.5), and dementia (CDR ≥ 1) groups. Only MCs but not NCs fulfilled the criterion of CDR ≥ 1. Thus, CDR ≥ 0.5 refers to the combination of CDR groups 0.5 and higher for the MCs compared to the CDR 0.5 NCs. Tables 1 and 2 show their demographic data. SMCs were assessed on Form B9 (Clinician Judgment of Symptoms: “Does the subject report a decline in memory?”) of the National Alzheimer's Coordinating Center (NACC) Uniform Data Set (UDS) [23]. The groups were subdivided by the presence or absence of SMCs. Estimated years to onset (EYO) were calculated as the age of the participant at assessment minus the age of the parent at symptom onset. EYO was given minus values in case of the years before estimated disease onset and plus values in case of the years after estimated disease onset. As all participants of the DIAN study are members of affected ADAD families, the construct of EYO can be applied to all (MCs and NCs), resulting in age-matched cases and controls. This construct of EYO has been validated in the DIAN study as providing a highly predictable clinical estimate of stage of AD biomarker profiles and symptom onset [18]. When the study was started, approximately 85% of participants did not know their mutation status. This recently has changed over the past one to two years with more participants having genetic counseling and testing. We estimated up to 40% now knowing their genetic status. However, the information which participant knows the mutation status is not available in the present database.

### 2.2. Measurement Methods

All demographical (age, gender, education, and estimated years to onset [EYO]), clinical (Geriatric Depression Scale [GDS]), and cognitive measurements (e.g., CDR global scores and sum of boxes [21, 22], logical memory subtest of The Wechsler Memory Test) were performed as recently described [18, 19].

### 2.3. Data Analysis

Subject characteristics were summarized as mean and standard deviation (SD) for continuous variables or number for categorical variables. Comparisons between the MCs and NCs on these characteristics were made with independent Mann-Whitney *U* tests or Chi-squared tests, respectively, Fisher's exact test in case of small expected cell counts (less than 5). The analyses of the overall association between memory complaints and CDR groups were performed using the Cochran-Armitage exact trend test, with Cramer's *V* used to gauge the relative magnitude of the association. For determination of diagnostic accuracy, we calculated the following characteristic numbers: accuracy, sensitivity, specificity, positive predictive value (PPV), and negative predictive value (NPV). Significance for the results was set at *P* < 0.05. All statistical analyses were performed using SAS 9.3 (SAS Institute, Inc., Cary, NC).

## 3. Results

### 3.1. Demographics of Participants of the DIAN Study

MCs and NCs were comparable regarding age and gender, showing no significant differences (Table 1). In addition, MCs had significantly lower educational levels, MMSE, and logical memory scores and higher CDR global scores, CDR sum of boxes, and GDS scores than their sibling NCs (Table 1).  Table 2 shows the characteristics of the participants of the DIAN study by CDR stage and carrier status. A comparison of the characteristics of the noncarriers (NCs) with CDR 0 versus CDR 0.5 is displayed in Table 3.

### 3.2. Percentage and Diagnostic Accuracy of SMCs in Participants of the DIAN Study Depending on Clinical Stage

**Figure 1:**
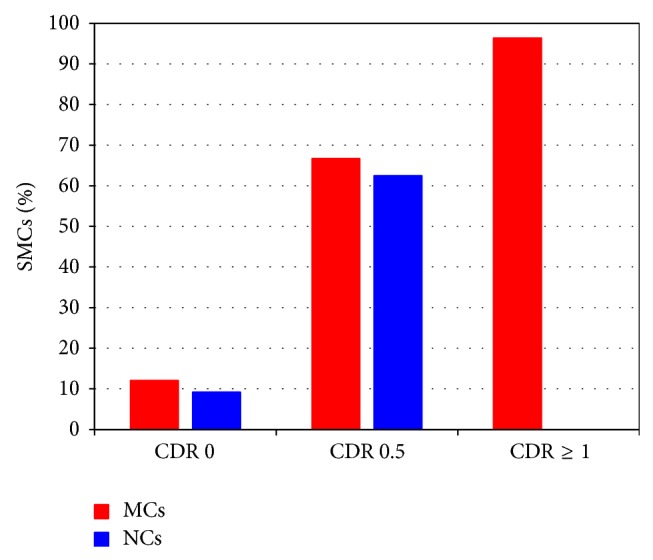
Percentage of mutation carriers (MCs) and noncarriers (NCs) with subjective memory complaints (SMCs) as a function of Clinical Dementia Rating (CDR) scale.

At CDR 0, SMCs were present in 12.1% of MCs and 9.2% of NCs (Chi-square test: *P* = 0.478). At CDR 0.5, SMCs were present in 66.7% of MCs and 62.5% of NCs (Fisher's exact test: *P* = 1.0). At CDR ≥1, SMCs were present in 96.4% of MCs (Figure 1).

Diagnostic accuracy of SMCs for being a MC at CDR 0 was 51.9% (sensitivity 12.2%, specificity 90.8%, PPV 56.5%, and NPV 51.3%), at CDR 0.5 62.5% (sensitivity 66.7%, specificity 37.5%, PPV 86.5%, and NPV 15.8%), and at CDR ≥ 0.5 (referring to the combination of CDR groups 0.5 and higher for the MCs compared to the CDR 0.5 NCs) 73.8% (sensitivity 77.6%, specificity 37.5%, PPV 92.2%, and NPV 15.0%).

### 3.3. Association between SMCs and Clinical Parameters in Participants of the DIAN Study

SMCs were positively correlated with CDR stages in MCs (Cramer's *V* = 0.687; trend test *P* < 0.0001) and in NCs (Cramer's *V* = 0.403; trend test *P* = 0.0008). SMCs were inversely correlated with logical memory scores in MCs (Cramer's *V* = −0.541; trend test *P* < 0.0001) but not in NCs (Cramer's *V* = −0.154; trend test *P* = 0.098). In addition, SMCs were positively correlated with EYO in MCs (Cramer's *V* = 0.609; trend test *P* < 0.0001), meaning that MCs with EYO <0 who were closer to onset (less years to onset) or MCs with EYO ≥0 who were farther from onset had a higher likelihood of SMCs but not in NCs (Cramer's *V* = 0.370; trend test *P* = 0.5809). Furthermore, SMCs were significantly inversely related to education in the cohort as a whole (Cramer's *V* = −0.175; trend test *P* = 0.0005) and in MCs (Cramer's *V* = −0.1352; trend test *P* = 0.0248) and NCs (Cramer's *V* = −0.2241; trend test *P* = 0.0341) separately. Moreover, SMCs were positively correlated with GDS scores in MCs (Cramer's *V* = 0.394; trend test *P* < 0.0001) and in NCs (Cramer's *V* = 0.197; trend test *P* = 0.033).

Using Spearman partial correlation, we found that SMCs in MCs were significantly correlated with logical memory scores even after controlling for GDS scores (*r* = −0.396; *P* < 0.0001). In NCs, SMCs showed a trend towards significance with logical memory after controlling for GDS (*r* = −0.179; *P* = 0.055).

Using linear mixed models, MCs with SMCs showed significantly lower logical memory scores than MCs without SMCs at CDR 0.5 (Mean ± Standard Error: 5.1 ± 0.8 versus 9.8 ± 1.1; *P* = 0.001) and at CDR ≥ 0.5 (8.3 ± 2.8 versus 9.2 ± 1.2; *P* < 0.0001) and a trend of significance at CDR 0 (10.9 ± 1.1 versus 13.3 ± 0.5; *P* = 0.066). In addition, MCs with SMCs showed significantly higher GDS scores than MCs without SMCs at CDR 0.5 (Mean ± Standard Error: 4.4 ± 0.7 versus 2.3 ± 0.6; *P* = 0.001) and at CDR ≥ 0.5 (8.4 ± 2.5 versus 2.2 ± 0.5; *P* < 0.0001) and a trend of significance at CDR 0 (2.3 ± 0.8 versus 1.5 ± 0.2; *P* = 0.083).

## 4. Discussion

The main findings of the present study were as follows. (1) Preclinical (asymptomatic) MCs of ADAD did not more frequently report SMCs in comparison with asymptomatic NCs. (2) The percentage and diagnostic accuracy of SMCs for being a MC increased with advancing cognitive impairment. (3) In MCs, SMCs were significantly positively associated with CDR, GDS, and EYO and inversely with education and logical memory scores.

In most current literature, the term “subjective cognitive decline” is used for defining a state in the preclinical stage of sporadic AD without objective cognitive impairment [17]. However, in the present study, the percentage of asymptomatic individuals with SMCs was rather low (≤12.1%) and did not differ between MCs and NCs. Thus, SMCs in asymptomatic members of ADAD families did not segregate with mutation status. This finding is in line with previous studies, showing that SMCs are not predictive of cognitive impairment or future cognitive decline [5–7]. A previous study with carriers of the PSEN1 E280A mutation identified a pre-MCI stage, characterized by memory complaints and coexisting objective cognitive impairment [20]. These findings indicate that SMCs in ADAD seem to occur rarely without additional objective cognitive impairment which is different from what is expected in sporadic AD [17]. This potential difference between ADAD and sporadic AD could be explained by a more aggressive course of ADAD [24] with an earlier onset of objective cognitive impairment and thus with a shorter asymptomatic phase.

The relevance of assessing SMCs is not restricted to preclinical AD, as nonspecialists in primary care are inaccurate at identifying dementia as well as MCI [25]. In the present study, frequency of SMCs clearly increased during the course of ADAD along with advancing cognitive impairment. According to a meta-analysis, SMCs were reported by 38% of those with known MCI and 43% of those with known dementia [16]. The higher percentage of SMCs in individuals with early symptomatic ADAD (66.7%) and dementia due to ADAD (96.4%) in the present study could be explained by the fact that members of families affected with ADAD may be more aware of or vigilant towards the cognitive changes than individuals in the general population. Additionally, the ADAD individuals are usually younger than those with late onset MCI/AD and therefore, any cognitive decline is more readily noticed without being ascribed to “age-related” limitations that can commonly be seen in older adults.

In the present study, 9.2% of asymptomatic NCs reported SMCs. This result is not surprising as in a recent study 14.4% of young adults (aged 18–39 years) reported SMCs [26]. Interestingly, 8/117 NCs showed in the present study a CDR of 0.5. Comparing the baseline characteristics in NCs with CDR 0.5 versus NCs with CDR 0 revealed that NCs with CDR 0.5 also showed higher CDR sum of boxes scores, a higher rate of SMCs, and a trend of lower education level compared to NCs with CDR 0.

Several previous studies have demonstrated a relationship between SMCs and depressive mood states [8–11]. In line with these findings, we also found positive correlations between SMCs and GDS scores in MCs and NCs. In addition, MCs with SMCs showed significantly higher GDS scores than MCs without SMCs at CDR 0.5 and at CDR ≥ 0.5 and a trend of significance at CDR 0. However, the significant relationship between SMCs and GDS does not rule out an ongoing neurodegenerative process or cognitive deterioration. We reported a significant association between SMCs, cognitive function (as measured by the logical memory subtest of the Wechsler Memory Test), and cognitive impairments (CDR results) as well as higher memory difficulties in MCs with SMCs. Interestingly, in MCs, we reported a significant association between subjective and objective measures of memory, even after controlling for depression effects (as measured using GDS). This significant association was not observed for NCs after controlling for depression score. These findings signify the sensitivity of SMCs to cognitive changes due to neurodegenerative processes of AD while their specificity requires further investigations.

A limitation of the study was that SMCs were captured by use of a single question with a binary threshold based on clinician judgement, which may be not sensitive enough to detect more subtle self-perceived changes. However, using a single-item question on SMCs has been previously reported to predict late onset AD incidence [3]. Another limitation was that the question concerning SMCs specifically referred to memory and did not include decline in behaviour, motor, or other cognitive functions. Furthermore, the number of individuals with SMCs in the preclinical stage of AD was rather small. In addition, the data analyzed were cross-sectional baseline data of the DIAN study, with limited predictive power concerning the EYO as opposed to individual longitudinal data. However, no sufficient longitudinal data are as yet available. It should also be noted that due to younger age, clinical observations resulted from studying ADAD may not be directly transferable to those in sporadic AD and vice versa.

## 5. Conclusions

Data of the present study indicate that SMCs assessed by a single-item scale have no diagnostic value to identify preclinical ADAD in asymptomatic individuals. These results demonstrate the need of further improvement of SMC assessment (at least in the ADAD population), for example, by additional use of multi-items and quantitative measures of SMCs. Additionally, multidomain cognitive complaint measures and inclusion of the informant part may increase the clinical utility of this construct. The diagnostic and clinical value of different SMC test instruments should be examined now in larger clinical trials with sporadic AD patients. The significant relationship of SMCs with logical memory and dementia stages, as we reported here, warrants further investigation into its clinical applications in general practice within elderly population.

## Figures and Tables

**Table 1:** Characteristics of the participants of the DIAN study.

Variables	Mutation carriers (MCs) (*N* = 183)	Noncarriers (NCs) (*N* = 117)	*P* value

Age (years)	39.1 ± 10.2	39.6 ± 10.3	0.6771^∗^
Gender (M/F)	81/102	47/70	0.485^∗∗^
Education level (years)	13.9 ± 2.7	14.6 ± 2.7	0.0343^∗^
CDR global	0.3 ± 0.6	0.03 ± 0.1	<0.0001^∗^
CDR sum of boxes	1.64 ± 3.2	0.1 ± 0.2	<0.0001^∗^
CDR 0 (number)	107	109	0.892^∗∗^
CDR 0.5 (number)	48	8	<0.0001^∗∗^
CDR ≥ 1 (number)	28	0	n.a.
Logical memory, delayed recall (percentile)	38.5 ± 36.8	60.9 ± 31.8	<0.0001^∗^
MMSE	26.8 ± 7.5	29.03 ± 1.3	<0.0001^∗^
GDS	3.8 ± 11.2	1.3 ± 1.6	<0.0001^∗^

^∗^Mann-Whitney *U* test; ^∗∗^Chi-square test; CDR denotes Clinical Dementia Rating Scale; M denotes male and F denotes female; MMSE denotes Mini-Mental Status Examination; GDS denotes Geriatric Depression Scale; age, education levels, CDR sum of boxes, MMSE, logical memory, and GDS scores are displayed in mean and standard deviation.

**Table 2:** Characteristics of the participants of the DIAN study by CDR stage and carrier status.

Variables	Mutation carriers (MCs) (*N* = 183)	Noncarriers (NCs) (*N* = 117)	*P* value

CDR 0			
Age (years)	35.1 ± 8.8	39.4 ± 10.2	0.002^∗^
Gender (M/F)	46/61	45/64	0.800^∗∗^
Education level (years)	14.2 ± 2.7	14.7 ± 2.7	0.092^∗^
CDR sum of boxes	0.03 ± 0.1	0.01 ± 0.08	0.186^∗^
Logical memory, delayed recall (percentile)	55.3 ± 33.2	62.2 ± 31.1	0.181^∗^
MMSE	29.0 ± 1.3	29.1 ± 1.3	0.488^∗^
GDS	1.6 ± 1.9	1.2 ± 1.5	0.129^∗^
SMCs (number)	13	10	0.478^∗∗^

CDR 0.5			
Age (years)	42.6 ± 9.3	42.4 ± 12.4	0.774^∗^
Gender (M/F)	21/27	2/6	0.449^∗∗∗^
Education level (years)	13.4 ± 2.3	12.5 ± 3.4	0.50^∗^
CDR sum of boxes	1.5 ± 1.1	0.6 ± 0.4	0.007^∗^
Logical memory, delayed recall (percentile)	17.4 ± 27.6	44.8 ± 39.4	0.015^∗^
MMSE	26.6 ± 2.8	28.4 ± 1.6	0.09^∗^
GDS	3.7 ± 3.4	2.1 ± 2.5	0.198^∗^
SMCs (number)	32	5	1.000^∗∗∗^

CDR ≥ 1			
Age (years)	48.4 ± 8.6	n.a.	
Gender (M/F)	14/14	0	
Education level (years)	12.8 ± 2.7	n.a.	
CDR sum of boxes	7.9 ± 4.1	n.a.	
Logical memory, delayed recall (percentile)	10.4 ± 27.1	n.a.	
MMSE	15.6 ± 6.3	n.a.	
GDS	4.1 ± 3.1	n.a.	
SMCs (number)	27	0	

^∗^Mann-Whitney *U* test; ^∗∗^Chi-square test; ^∗∗∗^Fisher's exact test; CDR denotes Clinical Dementia Rating Scale; M denotes male and F denotes female; MMSE denotes Mini-Mental Status Examination; GDS denotes Geriatric Depression Scale; age, education levels, CDR sum of boxes, MMSE, logical memory, and GDS scores are displayed in mean and standard deviation.

**Table 3:** Comparison of the characteristics of the noncarriers (NCs) with CDR 0 versus CDR 0.5.

Variables	Noncarriers (NCs) CDR 0 (*N* = 109)	Noncarriers (NCs) CDR 0.5 (*N* = 8)	*P* value

Age (years)	39.4 ± 10.2	42.4 ± 12.4	0.552^∗^
Gender (M/F)	45/64	2/6	0.427^∗∗^
Education level (years)	14.7 ± 2.7	12.5 ± 3.4	0.068^∗^
CDR sum of boxes	0.01 ± 0.08	0.6 ± 0.4	<0.0001^∗^
Logical memory, delayed recall (percentile)	62.2 ± 31.1	44.8 ± 39.4	0.257^∗^
MMSE	29.1 ± 1.3	28.4 ± 1.6	0.125^∗^
GDS	1.2 ± 1.5	2.1 ± 2.5	0.285^∗^
SMCs (number)	10	5	0.001^∗∗^

^∗^Mann-Whitney *U* test; ^∗∗^Fisher's exact test; CDR denotes Clinical Dementia Rating Scale; M denotes male and F denotes female; MMSE denotes Mini-Mental Status Examination; GDS denotes Geriatric Depression Scale; age, education levels, CDR sum of boxes, MMSE, logical memory, and GDS scores are displayed in mean and standard deviation.
